# Apomictic *Malus* plants exhibit abnormal pollen development

**DOI:** 10.3389/fpls.2023.1065032

**Published:** 2023-02-20

**Authors:** Dan-Dan Liu, Da-Ru Wang, Xuan-Yu Yang, Chang-Hui Zhao, Shao-Hua Li, Guang-Li Sha, Rui-Fen Zhang, Hong-Juan Ge, Xian-Song Tong, Chun-Xiang You

**Affiliations:** ^1^ College of Agriculture, Yunnan University, Kunming, Yunnan, China; ^2^ National Key Laboratory of Crop Biology, National Research Center for Apple Engineering and Technology, College of Horticulture Science and Engineering, Shandong Agricultural University, Tai-An, Shandong, China; ^3^ Qingdao Academy of Agricultural Sciences, Qingdao, Shandong, China; ^4^ Fu-ning Popularizing Agricultural Techniques Center, Fu-ning, Yunnan, China

**Keywords:** apple, apomixis, transcriptome analysis, pollen abortion, apomeiosis

## Abstract

Apomixis is the asexual reproduction through seeds that leads to the production of genetically uniform progeny. It has become an important tool in plant breeding because it facilitates the retention of genotypes with desirable traits and allows seeds to be obtained directly from mother plants. Apomixis is rare in most economically important crops, but it occurs in some *Malus* species. Here, the apomictic characteristics of *Malus* were examined using four apomictic and two sexually reproducing *Malus* plants. Results from transcriptome analysis showed that plant hormone signal transduction was the main factor affecting apomictic reproductive development. Four of the apomictic *Malus* plants examined were triploid, and pollen was either absent or present in very low densities in the stamen. Variation in the presence of pollen was associated with variation in the apomictic percentage; specifically, pollen was absent in the stamens of tea crabapple plants with the highest apomictic percentage. Furthermore, pollen mother cells failed to progress normally into meiosis and pollen mitosis, a trait mostly observed in apomictic *Malus* plants. The expression levels of meiosis-related genes were upregulated in apomictic plants. Our findings indicate that our simple method of detecting pollen abortion could be used to identify apple plants that are capable of apomictic reproduction.

## 1 Introduction

Apomictic seeds are the products of asexual reproduction and give rise to maternal clones; meiosis and double fertilization are not required for the production of apomictic seeds (Hand and Koltunow, 2014). Apomixis results in the production of genetically identical offspring. It has become an important breeding tool in agriculture because it permits the generation of clones of mother plants as well as the retention of crop genotypes with desirable features. Studies of apomixis have been conducted for over 150 years. Female and male heteromorphic plants of *Achornea ilicifolia* were shown to produce seeds without pollination by Smith in 1841 (Smith, 1841). Apomixis has been documented to occur in over 400 species in 61 families, including Poaceae, Rosaceae, and Asteraceae. Apomictic reproduction is not widespread in crops, but it is common in horticultural crops, such as citrus, crabapple, walnut, mango, and Chinese chive (Cosmulescu et al., 2012; Yamashita et al., 2012; Liu et al., 2014; Kuhn et al., 2017; Wu et al., 2021).

In apomictic plants, female gametes are formed without meiosis (apomeiosis) and the embryo develops without fertilization; the formation of functional endosperm is also mediated by several developmental adaptations in apomictic plants (Koltunow and Grossniklaus, 2003; Hao and Liu, 2011). Apomixis can be classified as obligate or facultative depending on whether plants can reproduce sexually. Apomixis can also occur *via* diplospory, apospory, and adventitious embryo reproduction depending on the developmental origin of apomictically derived embryos. Diplospory and apospory are both forms of gametophytic apomixis, wherein unreduced cells give rise to a megagametophyte *via* mitosis, and adventitious embryo reproduction is a form of sporophytic apomixis, wherein unreduced cells give rise directly to an embryo (Tucker and Koltunow, 2009). Apomixis, especially facultative apomixis, is common in apple trees in the family Rosaceae, and natural pollination often leads to the production of a small number of sexual hybrids (Liu et al., 2014).

Several apomixis-related genes involved in the regulation of embryo sac genes have been identified in *Arabidopsis thaliana*, petunia, maize, and other plants in recent years. For example, *SWITCH1/DYAD* promotes female gametophyte formation by causing cells to bypass meiosis (Mercier et al., 2001; Mercier et al., 2003). *SOMATIC EMBRYOGENESIS RECEPTOR KINASE 1* (*SERK1*), *LEAF COTYLEDON 1* (*LEC1*), *LEC2*, and *BABYBOOM* (*BBM*) have been shown to induce somatic embryogenesis in plants (Boutilier et al., 2002; Ranganath, 2004; Santos and Aragão, 2009). In *Pliosella*, which is a subgenus of *Hieracium*, apomixis and the autonomous development of seeds are regulated by two independent dominant loci, *LOSS OF APOMEIOSIS* (*LOA*) and *LOSS OF PARTHENOGENESIS* (*LOP*), respectively (Catanach et al., 2006; Okada et al., 2007; Koltunow et al., 2011). *CitRWP*, which contains an RWP-RK domain, regulates polyembryonic characters and various morphological aspects of the plant body during citrus embryogenesis (Wang et al., 2017; Wang et al., 2022). In *Zanthoxylum bungeanum*, the expression of *AGL11* is correlated with nucellar embryo development, and the ectopic expression of this gene can result in abnormal flower development and apomixis phenotypes in *Arabidopsis* (Fei et al., 2021). Various studies have attempted to genetically engineer plants with gametophytic apomixis. The *MiMe* (Mitosis instead of Meiosis) system was first created in *Arabidopsis via* the simultaneous mutation of three key meiotic genes (*SPO11-1*, *REC8*, and *OSD1*). *MiMe*, coupled with the expression of *BBM1* (a parthenogenesis gene) in the egg cell, can also induce the production of clonal progeny in hybrid rice and mediate the retention of genome-wide parental heterozygosity (Khanday et al., 2019). Nevertheless, there are still several shortcomings associated with synthetic apomixis.

Apomictic reproduction has been documented in a total of 10 *Malus* species (Shu, 1999; Li, 2001). Six of these species are native to China, and these species comprise a group containing 23 species (e.g., *Malus hupehensis*, *Malus sikkimensis*, *Malus rockii*, *Malus sieboldii*, *Malus platycarpa*, *Malus sargentii*, *Malus xiaojinensis*, *Malus lancifolia*, and *Malus corolla*). Most angiospermous show facultative apomixis because they can set seeds both sexually and apomictically depending on their genotype and environmental conditions (Carman et al., 2011; Liu et al., 2014). Apospory is widespread among polyploid species in the genus *Malus* (e.g., tea crabapple) (Schmidt, 1977). In apospory, the embryo sac develops directly from ovule cells and the embryo develops from the unreduced egg cells (Liu et al., 2014). Tea crabapple (*M. hupehensis* var. *pingyiensis*) is facultatively apomictic, and both parthenogenesis and apomeiosis have been documented in this species. The apomictic capacity of this species is controlled by separate genetic loci, and these loci affect both apomeiosis and parthenogenesis (Liu et al., 2014).

Much research has focused on developing approaches to facilitate the production of apomictic seeds and produce seedlings with uniform characteristics from apomictic crabapple rootstocks. Although much progress has been made in the genetic engineering of *Malus* species, there are still many challenges that need to be overcome for the genetic improvement of apomictic *Malus* species. A deep understanding of the mechanism of apomixis is essential for ensuring that genetic modifications have their desired effect. Tea crabapple, which is referred to as PYTC in this study, is a triploid plant that is facultatively apomictic; it is often used as a rootstock in production. Previous analysis of the development and genetic characteristics of apomixis in PYTC has shown that the bypassing of pollination and fertilization is mainly achieved through a delay in embryo sac development (female late-in-date), and this mediates the production of asexual seeds (Liu et al., 2014). Here, we evaluated the apomictic capacity of PYTC and then characterized several genes involved in facultative apomixis by conducting a comparative transcriptomic analysis of the flowers of a hybrid population. The reference transcriptome generated in our study will aid future studies aimed at elucidating the regulatory networks and pathways involved in apomixis in *Malus*. We also evaluated the apomictic capacity in *Malus*; most of the material examined was triploid, and pollen development was abnormal. Overall, our study provides a simple method that could be used to identify apomictic apple rootstocks.

## 2 Materials and methods

### 2.1 Plant materials

Apomictic crabapple species examined in our study included the triploid tea crabapple (*M. hupehensis* Redh. var. *pingyiensis*, PYTC, 2n=3X=51, facultatively apomictic), triploid *M. sargentii* (SJHT, 2n=3X=51, facultatively apomictic), *M. sikkimensis* (SYHT, 2n=3X=51, facultatively apomictic), and *M. sieboldii* (XJHT, 2n=3X=51, facultatively apomictic); both *Malus domestica* “Jonagold” (QNJ, 2n=3X=51, triploid but can reproduce sexually) and *Malus baccata* (SDZ, 2n=3X=34, diploid and can reproduce sexually) were used as controls. All plants were grown in the experimental orchard of Shandong Agricultural University and Yunnan University.

### 2.2 Evaluation of apomixis and estimation of ploidy

The apomictic capacity of the crabapple species examined was evaluated following the methods of Liu et al. (2014). To determine the parthenogenesis percentage, pistils were decapitated in the spring before the flowers opened. The parthenogenesis percentage was calculated as the number of parthenocarpic fruits that could produce seeds in the absence of fertilization relative to the number of decapitated flowers. To determine the apomeiosis percentage, asexual seeds were harvested from parthenocarpic fruits. The apomeiosis percentage was calculated as the number of triploid seeds relative to the total number of seeds tested.

Ploidy was determined using flow cytometry assays, which were conducted using the Partec CyStain UV Precise T reagent kit (PARTEC, Cod. 05-5003) per the manufacturer’s instructions. First, young leaves or embryos from mature seeds were cut into smaller pieces with a razor blade in nuclei extraction buffer. Staining buffer was then added with the one-fifth volume of nuclei extraction buffer following the manufacturer’s instructions. Next, debris was removed, and nuclei were collected by filtering the nuclear suspensions two times through a 20-µm nylon mesh. An arc lamp-based flow cytometer (PARTEC PA, Germany) was used to measure the fluorescence intensity of the nuclei. The fluorescence intensity of 30,000 nuclei per cytogram was measured. At least three different plants for each line were measured, and each sample was analyzed in triplicate.

### 2.3 Morphological observations and pollen viability experiment

Morphological observations of the anthers were made by taking photographs with a Nikon SMZ18 microscope, and a scalpel was used to dissect the anthers after the photographs were taken.

The anthers were peeled, cut open, and placed on a slide, and forceps were used to extract the pollen grains. Two to three drops of I_2_–KI solution were added; after mixing thoroughly, the slide was covered with a coverslip for staining. One of the potassium iodide was then dissolved in a small amount of distilled water. After the potassium iodide and distilled water were fully dissolved, 0.5 g of iodine was added. The mixture was then shaken to dissolve all the components; it was then diluted to 150 ml and stored in a brown glass bottle.

### 2.4 Histological analysis

Anthers were fixed in formaldehyde–alcohol–acetic acid fixative (90 ml of 70% alcohol + 5 ml of formalin + 5 ml of glacial acetic acid). Paraffin sectioning was conducted following the method described in a previous study to characterize the histology of the fixed samples (Yang et al., 2013).

### 2.5 RNA sequencing and data analysis

The Trizol kit (Invitrogen, USA) was used to extract total RNA from all the plant materials, and the RNA sequencing (RNA-seq) library was constructed using the RNA obtained. After sequencing, the raw data were filtered to obtain high-quality clean reads. Differentially expressed genes (DEGs) were identified using the DEGseq R package with the following criteria: false discovery rate (FDR) < 0.001 and |log_2_(fold change (FC))| ≥ 1. The GOSeq R software package (*p*-value ≤ 0.05) was used to conduct a Gene Ontology (GO) analysis of the DEGs.

### 2.6 qRT-PCR assays

An ABI 7500 Real-time PCR system was used to conduct qRT-PCR analyses of the extracted RNA following the procedures described in a previous study (Wang et al., 2022). The 2^−ΔΔCt^ method was used to determine expression levels. Three biological replicates were performed for each sample. **Supplementary Table 1** shows the primers that were used in qRT-PCR analyses.

### 2.7 Statistical analysis

Data processing system software was used to analyze the data (Wang et al., 2022). The significance of differences between groups was evaluated using a one-way analysis of variance (ANOVA), followed by the Tukey–Kramer test; the threshold for statistical significance was *p*-value < 0.05.

## 3 Results

### 3.1 The apomictic capacity of *Malus* plants varied

**Figure 1:**
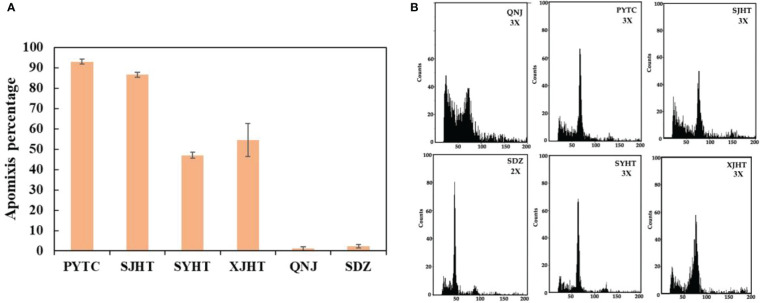
Apomictic percentage and ploidy analysis of different *Malus* plants. **(A)** Apomictic percentage analysis. **(B)** Ploidy analysis of apomictic *Malus* plants.

The apomictic percentages of *Malus* plants were evaluated from 2019 to 2021. The lowest apomictic percentages were observed for QNJ (1.3%) and SDZ (2.8%). The apomictic percentages of PYTC, SJHT, SYHT, and XJHT were 94.7%, 86.1%, 41.5%, and 52.7%, respectively (**Figure 1A**). The apomictic percentage of PYTC was the highest among all apple species. Additionally, ploidy analyses revealed that SDZ was diploid, and PYTC, SJHT, SYHT, XJHT, and QNJ were triploid (**Figure 1B**), suggesting that all detected apomictic *Malus* plants were polyploid. Nonetheless, the sexual line QNJ was also found to be polyploid, suggesting that polyploidy may not be the only driving factor leading to apomixis.

### 3.2 DEGs in *Malus* with high and low apomictic percentages

Plants in the genus *Malus* are cross-pollinated, and the progeny show high heterozygosity. Hybrid progeny were obtained *via* artificial pollination, and these hybrid progeny populations were used to clarify the mechanism underlying apomixis in *Malus*. Apomictic reproduction is performed facultatively in tea crabapple (PYTC) in its natural environment, and PYTC exhibits the highest apomictic percentage among all *Malus* plants. Sexually reproducing hybrids were obtained *via* artificial pollination of PYTC pistils with pollen from another wild apple species (*Malus pumila* Mill. var. “*Maypole*”). Floral development comprises six stages (Liu et al., 2014). The ovules of hybrids derived from tea crabapple and *Maypole* were collected at stage 2, which is the pre-meiotic development stage, from two populations differing in parthenogenesis percentages: P1 (21.3 ± 1.6%; low parthenogenesis percentage) and P2 (76.7 ± 5.3%; high parthenogenesis percentage) (Liu et al., 2014).

**Figure 2:**
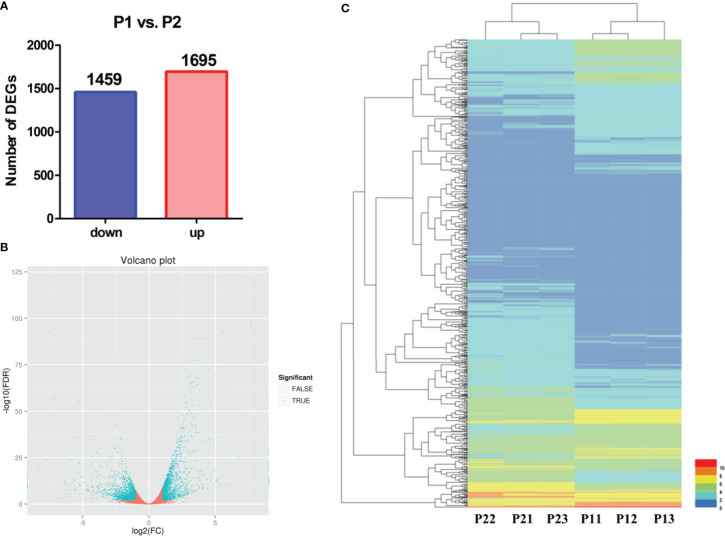
Preliminary analysis of transcriptomic data from P1 and P2. **(A)** Statistical analysis of downregulated and upregulated DEGs in the P1 *vs*. P2 comparison group. **(B)** Volcano plots of DEGs in the P1 *vs*. P2 comparison group. **(C)** Heatmap of the expression levels of DEGs in the P1 *vs*. P2 comparison group.

To identify apomixis-related genes in *Malus*, RNA-seq analysis was conducted on P1 and P2. Overall, a total of 24,126,591 reads were obtained in P1, and a total of 22,785,112 reads were obtained in P2. In P1, the GC content was 47.11%, the total number of clean reads obtained was 22,785,112, and the total number of bases was 7,237,977,400; in P2, the GC content was 46.89%, and the total number of bases was 6,835,533,600 (**Supplementary Table 2**). A total of 3,154 DEGs were identified using the following criteria: FDR < 0.001 and |log_2_(FC) ≥ 1|. The expression of 1,459 and 1,695 DEGs was downregulated and upregulated, respectively (**Figure 2A**). A volcano plot was constructed to visualize variation in the number of DEGs among comparison groups. The differential expression of genes was higher the closer they were located at the ends of the volcano plot (**Figure 2B**). Cluster analysis was conducted on the DEGs (**Figure 2C**).

### 3.3 Functional classification of DEGs

**Figure 3:**
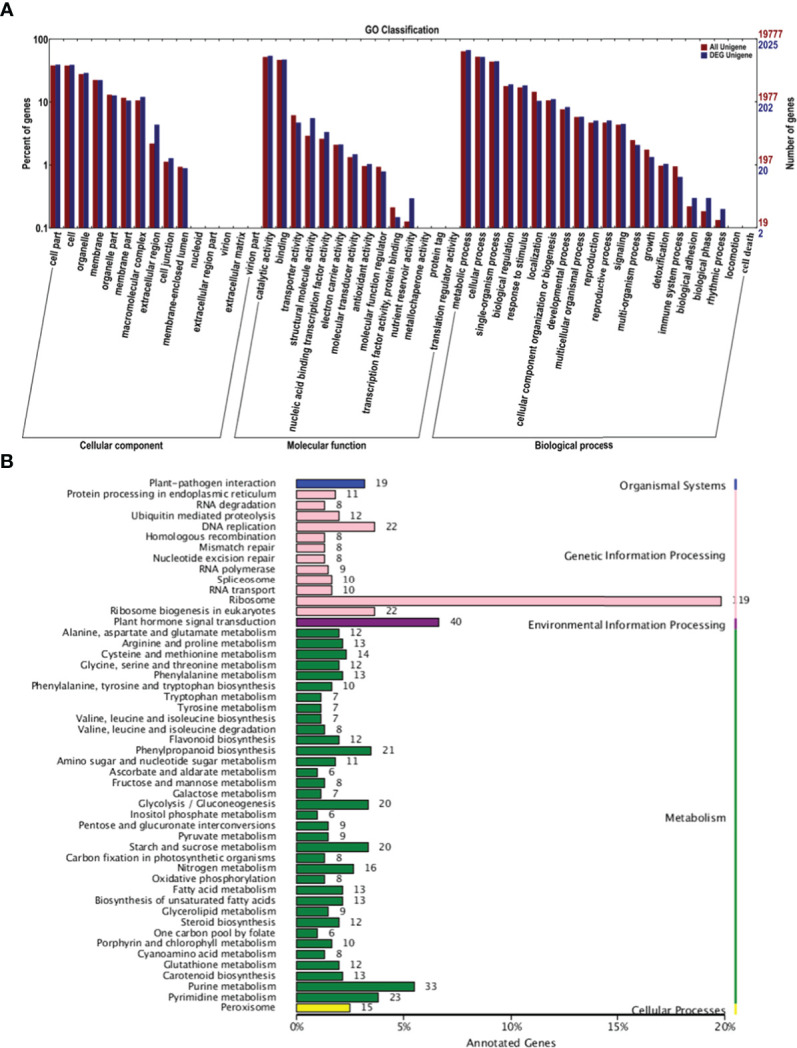
GO and KEGG analysis of DEGs in the P1 *vs*. P2 comparison group. **(A)** GO analysis of DEGs in the P1 *vs*. P2 comparison group. **(B)** KEGG analysis of DEGs in the P1 *vs*. P2 comparison group.

GO analysis was conducted to characterize the functions of DEGs in P1 and P2 (**Figure 3A**). DEGs were assigned GO terms in three categories: cellular component (CC), molecular function (MF), and biological process (BP). In the CC category, DEGs were mainly enriched in the following GO terms: “cell part,” “cell,” and “organelle.” In the MF category, DEGs were mainly enriched in the following GO terms: “catalytic activity” and “binding.” In the BP category, most DEGs were enriched in the following GO terms: “metabolic process,” “cellular process,” and “single-organism process.” Most of these processes are involved in pollen development.

Kyoto Encyclopedia of Genes and Genomes (KEGG) analysis was conducted on the DEGs to further clarify their functions. The main pathways were divided into the following categories: organismal systems, genetic information processing, environmental information processing, metabolism, and cellular processes (**Figure 3B**). In genetic information processing, the most enriched pathways were “ribosome,” “DNA replication,” and “ribosome biogenesis in eukaryotes.” The most enriched pathway in environmental information processing was “plant hormone signal transduction.” The most enriched pathway in metabolism was “purine metabolism.” These findings suggest that hormone signal transduction played a role in regulating apomixis.

### 3.4 Pollen development was abnormal in apomictic *Malus* plants

**Figure 4:**
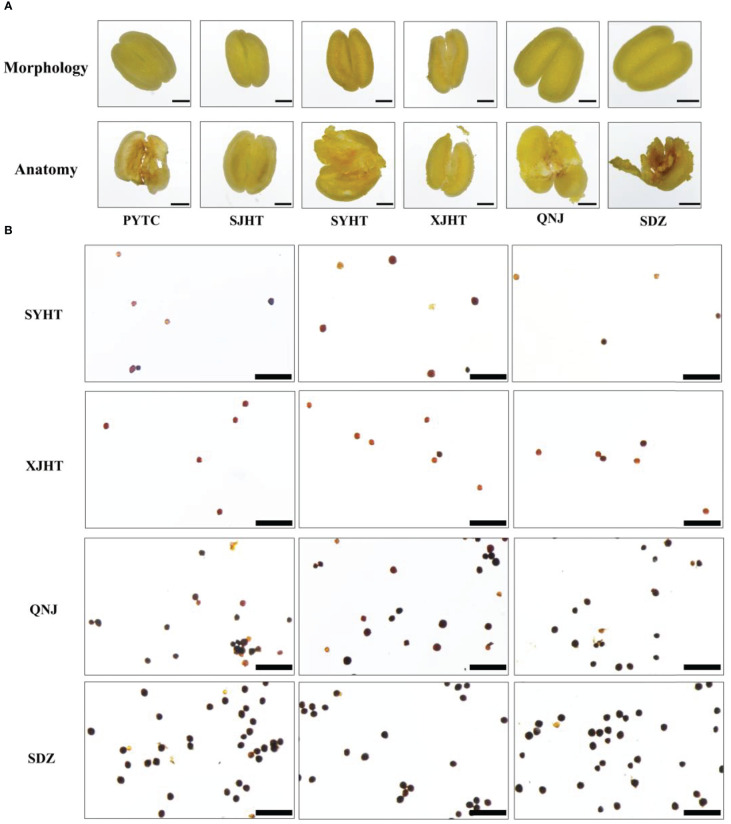
Morphological observations of anthers and evaluation of the activity of pollen grains in apple plants. **(A)** Morphological structure of the anthers of PYTC, SJHT, SYHT, XJHT, QNJ, and SDZ and the presence of pollen after dissection. Bar, 500 μm. **(B)** Pollen viability assays for SYHT, XJHT, QNJ, and SDZ. Bar, 200 μm.

All detected apomictic *Malus* plants were polyploid. We studied the pollen development process and measured pollen viability in apomictic *Malus* plants to determine whether pollen development was affected by polyploidy. No pronounced differences in anther morphology were observed between apomictic and sexually reproducing *Malus* plants. Pollen was absent in the anthers of PYTC and SJHT, which were the two *Malus* species with the highest apomictic percentage (**Figure 4A**). In addition, the abundance of pollen in the apomictic *Malus* species SYHT and XJHT was less than that in the sexually reproducing species QNJ and SDZ. The activity of pollen grains in SYHT, XJHT, QNJ, and SDZ was evaluated *via* I_2_–KI staining (**Figure 4B**). The amount of pollen was significantly lower in each field of SYHT and XJHT than in QNJ and SDZ. Most of the pollen grains in QNJ and SDZ were stained blue or blue-black, suggesting that the degree of starch accumulation was high in QNJ and SDZ. By contrast, most of the pollen grains in SYHT and XJHT were stained yellow or yellow-brown, and only a few were stained blue or blue-black. This suggests that starch accumulated in only a few of the pollen grains and thus that most of the pollen grains were not viable pollen.

### 3.5 The expression patterns of genes involved in pollen development are altered in apomictic *Malus* plants

**Figure 5:**
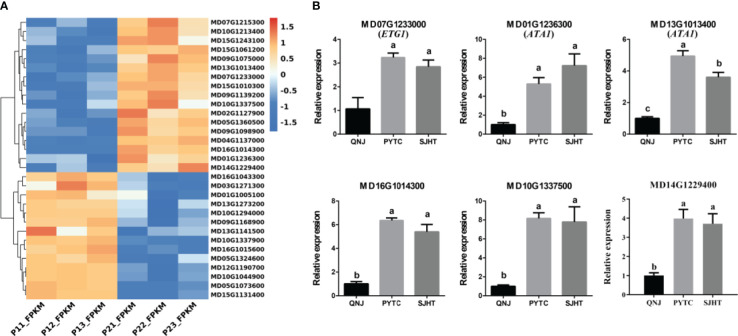
Analysis of DEGs involved in pollen development in the P1 *vs*. P2 comparison group. **(A)** Cluster heatmap of genes involved in pollen development in the P1 *vs*. P2 comparison group. **(B)** The expression patterns of six of the DEGs in **(A)** were analyzed by qRT-PCR. Groups with different lowercase letters are significantly different according to ANOVA, followed by the Tukey–Kramer test (*p* < 0.05). Data are the mean ± standard deviation of three independent replicates.

We identified genes involved in pollen development using the RNA-seq data to clarify the relationship between pollen development and apomixis (**Figure 5A**). We identified a total of 31 significant DEGs in the P1 *vs*. P2 comparison group. Next, qRT-PCR analyses were conducted to measure the expression levels of six genes (*MD16G1014300*, *MD07G1233000*, *MD01G1236300*, *MD13G1013400*, *MD10G1337500*, and *MD14G1229400*) in QNJ, PYTC, and SJHT (**Figure 5B**). The homologous gene of *MD07G1233000* (*ETG1*) in *Arabidopsis* plays a key role in the establishment of sister chromatid cohesion in mitosis and meiosis. *MD01G1236300* and *MD13G1013400* (*ATA1*) may regulate the development of the tapetum, which provides nutrients for the development of pollen. *MD16G1014300* regulates the development of flowers, *MD10G1337500* encodes a heavy metal transport/detoxification superfamily protein, and *MD14G1229400* plays a key role in the development of the cytoplasm of pollen. The expression of all six genes was lower in QNJ than in PYTC and SJHT, and differences in the expression of this gene among *Malus* species were consistent with differences in the apomictic percentage. These findings suggest that there is a relationship between apomixis and pollen development.

### 3.6 Meiotic abnormalities result in the premature termination of pollen development in apomictic *Malus* plants

**Figure 6:**
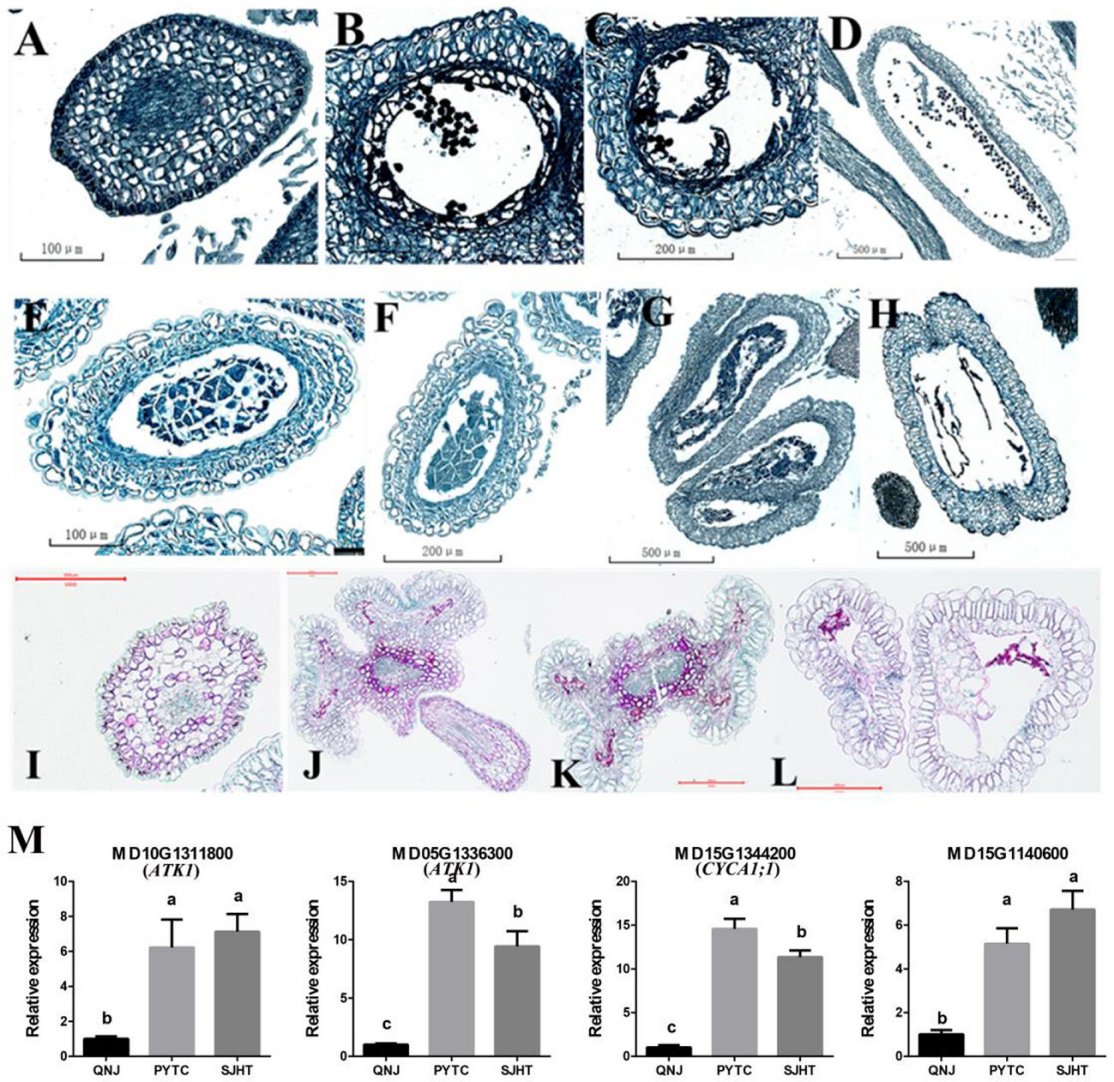
Paraffin sectioning and quantification of the expression of genes involved in meiosis at different stages of pollen development in the anthers of QNJ, PYTC, and SJHT. **(A–D)** QNJ anthers at different developmental stages. **(A)** Microspore mother cell stage; **(B)** early uninucleate stage; **(C)** late uninucleate stage; and **(D)** mature stage. **(E–H)** PYTC anthers at different developmental stages. **(E, F)** Microspore mother cell stage; **(G)** callose degradation; **(H)** and mature stage. **(I–L)** SJHT anthers at different developmental stages. **(I)** Microspore mother cell stage; **(J, K)** callose degradation; **(K, L)** mature stage. **(M)** The expression levels of meiosis-related genes were determined by qRT-PCR analyses. Groups with different lowercase letters are significantly different according to ANOVA, followed by the Tukey–Kramer test (*p* < 0.05). Data are the mean ± standard deviation of three independent replicates.

Paraffin sectioning was used to identify the stage at which pollen development ceases and determine why pollen development was abnormal in apomictic *Malus* plants. Pollen development in PYTC and SJHT ceased at the meiosis stage of pollen mother cells (**Figures 6F, I**), and defects in tapetal development were the ultimate cause of pollen abortion. No mature pollen grains were detected in the anthers at the final maturation stage (**Figures 6H, L**). Pollen development was normal in the sexually reproducing QNJ (**Figures 6A–D**).

Pollen development did not proceed normally in PYTC and SJHT because of the failed meiosis of microspore mother cells. To identify candidate genes that contribute to the observed meiotic abnormalities in PYTC and SJHT, we measured the expression levels of some of the meiosis-related genes (**Supplementary Figure 1**). Genes *MD10G1311800* and *MD05G1336300* may be homologs of *Arabidopsis ATK1*, a gene that plays a key role in the assembly of the spindle during male meiosis and may code for a kinesin, while *MD15G1344200* may correspond to *Arabidopsis CYCA1;1*, a gene that may play a role in the mitotic cell cycle phase transition, and *MD15G1140600* negatively regulates cell biosynthesis. We also found that the expression of these four genes was higher in PYTC and SJHT than in QNJ (**Figure 6M**). The differential expression of these genes might contribute to the abnormal meiosis of PYTC and SJHT.

## 4 Discussion

Generally, plants undergo sexual reproduction to form seeds. However, some plants have evolved alternative modes of reproduction that allow them to bypass the meiotic and double fertilization processes of sexual reproduction before seed formation. This process of asexual seed production is called apomixis (Bicknell and Koltunow, 2004). Several studies have attempted to generate apomictic plants *via* genetic approaches, but no breakthroughs in the generation of apomictic plants have yet been made (Leblanc et al., 2009; Rodriguez-Leal and Vielle-Calzada, 2012). One of the major challenges is that apomixis is a highly complex trait, and apomeiosis, parthenogenesis, and developmental adaptation are required for the formation of the functional endosperm. Several genes are involved in regulating each of these processes (Koltunow and Grossniklaus, 2003). Differences in the expression of plant hormone signaling genes have been observed between sexually reproducing and apomictic *Arabidopsis* and *Paspalum notatum* (Figueiredo and Köhler, 2018; Ortiz et al., 2019). In this study, transcriptome analysis of the hybrid progeny of PYTC revealed that hormone signal transduction was the main factor affecting apomictic reproductive development, and additional studies are needed to clarify the relative importance of apomixis in *Malus* plants.

Nearly all asexually reproducing plants (including both herbaceous and woody plants) identified to date are polyploid (Sax, 1959). All the *Malus* plants in our study were polyploid, and the apomictic percentage of these plants varied, indicating that there might be a relationship between apomixis and polyploidy (**Figure 1**). PYTC, SJHT, SYHT, and XJHT were all triploid and had high apomictic percentages; SDZ, which does not reproduce *via* apomixis, was diploid. Although QNJ was triploid, it is not capable of apomictic reproduction, suggesting that genes related to apomixis have been lost in this apple species.

Pollen was absent in the anthers of PYTC and SJHT (**Figure 4A**). The amount of pollen in the anthers of SYHT and XJHT was low (**Figure 4B**). Analysis of the expression of genes involved in pollen development revealed that the expression of these genes in PYTC and SJHT significantly differed from their expression patterns in QNJ (**Figure 5B**). Pollen development is a complex process, and abnormalities in meiosis, the tapetal layer, and pollen sac formation can result in abortive or abnormal development (Jeong et al., 2014; Wu et al., 2015; Somaratne et al., 2017; Zhao et al., 2018; Yang et al., 2019). In apomictic *Malus* plants, pollen development ceased at the meiosis stage, which suggests that apomeiosis failed in pollen development. Thus, pollen arrest could be used to identify apomictic plants; however, polyploidy does not necessarily result in pollen abortion. In Melastomataceae species, apomictic plants with no viable pollen or with pollen with low viability do not receive visits from pollinators and likely produce strictly apomictic fruits, whereas apomictic and sexually reproducing plants with high pollen viability might produce fruits and seeds through both sexual and apomictic reproduction (Maia et al., 2016). Pollen viability levels are important determinants of flower visitation regardless of whether plants reproduce sexually or apomictically. In *Malus* plants, pollen viability seems linked to a high occurrence of apomixis.

Polyploidy and hybridization might induce shifts in mechanisms controlling reproduction, and the repatterning of gene expression might result in the evolution of gametophytic apomixis (Carman, 2001; Grimanelli et al., 2001; Carman, 2007). Pollen development in plants is a complex process that requires meiosis, the division of haploid nuclei, haploid nuclear division, cellular differentiation of male gametophytes, and other processes (Goldberg et al., 1993; Hafidh et al., 2016; Liu and Wang, 2021). In diploid plants, pollen is formed *via* the meiosis and mitosis of pollen mother cells. We conducted a transcriptome analysis to clarify the relationship between apomixis and pollen development in a population with a low apomictic percentage (P1) and a population with a high apomictic percentage (P2). KEGG analysis revealed DEGs that were significantly enriched in DNA replication, ribosome, plant hormone signal transduction, and purine metabolism (**Figure 3B**). This suggests that P2 plants show abnormalities in meiosis and hormone metabolism during pollen development and metabolism. Many studies have shown that there is a close relationship between hormone metabolism and pollen development. Auxin levels in pollen grains affect the normal development of stamens and anthers in *Arabidopsis* (Salinas-Grenet et al., 2018). The programmed death of microspore mother cells during meiosis is partly induced by abscisic acid and indole-3-acetic acid (Kovaleva et al., 2018).

To further clarify the causes of pollen abortion, we studied the pollen development of QNJ, PYTC, and SJHT using paraffin sectioning (**Figures 6A–L**). We found that meiosis failed in pollen mother cells in PYTC and SJHT. Meiosis is a critically important stage in the process of pollen development, and meiotic abnormalities can result in pollen abortion. Several genes are involved in the regulation of meiosis. In *Arabidopsis*, approximately 50 genes have been shown to directly regulate meiosis, and many have been shown to indirectly regulate meiosis (Sanchez-Moran and Armstrong, 2014). We found that the transcript level of the meiosis-related genes *MD05G1336300* and *MD15G1344200* in *Malus* plants was upregulated in PYTC and SJHT, leading to abnormal division of pollen mother cells, suggesting that meiotic abnormalities were the cause of abnormal pollen development. The regulation of meiosis in *Malus* species might be more complex given that they are perennial woody plants.

## 5 Conclusion

In sum, *Malus* plants that reproduce apomictically may be identified by determining whether the morphology of pollen is abnormal and its viability is low. Thus, the methods used in our study may facilitate the identification of apple plants that are capable of apomictic reproduction. Further characterization of genes involved in pollen mother cell division such as *MD05G1336300* (*ATK1*) and *MD15G1344200* (*CYCA1;1*) may improve our understanding of meiosis in apples for breeding purposes.

## Data availability statement

The original contributions presented in the study are included in the article/**Supplementary Material**. Further inquiries can be directed to the corresponding authors.

## Author contributions

Data curation, D-RW. Formal analysis, X-YY, C-HZ and S-HL. Funding acquisition, D-DL and C-XY. Investigation, D-DL and C-XY. Resources, G-LS, R-FZ and H-JG. Visualization, D-RW and X-ST. Writing—original draft, D-RW and D-DL. All authors contributed to the article and approved the submitted version.
