# Supplementary material for: Apomictic Malus plants exhibit abnormal pollen development
Source: Front Plant Sci. 2023 Feb 20;14:1065032. doi: 10.3389/fpls.2023.1065032 (PMC9986266; doi:10.3389/fpls.2023.1065032)
Supplement: Supplementary file 1 [file DataSheet_1.docx]

**Supplementary Materials:**

**Table S1:** Primers for quantitative real-time PCR.

| **Gene** | **Forward primer (5′-3′)** | **Reverse primer (5′-3′)** |
| --- | --- | --- |
| MD16G1014300 | GCAACCCAGCTTATGCAACC | GAGAGGGAGATCGGGCATTG |
| MD07G1233000 | TATGGGAACGCCGTATGCTC | TCTCGGGCGCTTCTCTTTTT |
| MD01G1236300 | TGGCATTTGCAAAGGAACCG | GCCGGATTGGTTATAGCCGA |
| MD13G1013400 | TCGAAGAGGAGGGAGCATCA | CATCGACGAGCATCTCCGAA |
| MD10G1337500 | CAGTGGTTATGGTGGGGGAC | GACTGTGCAAGCTGAGGGAT |
| MD14G1229400 | GGATGTCCCGCTTGAAGGAA | TTTCGCAGAAGCCAACAAGC |
| MD10G1311800 | ATCGCCTCGCTGAATCAGAG | AACTACAGGGGCGTCTGTTG |
| MD05G1336300 | AGTTCCCTTAGACAAGCGGC | ACGCCTCGACTTCTTCCTTG |
| MD15G1344200 | AAGGGGCCAATGAGGTTACG | TGGCATTCCACGGTCTCTTC |
| MD15G1140600 | CCCAATTTCCAGGAGCGACT | CTCCATTCTTCCGCTGGTGT |

**Table S2:**

| **Population** | **Reads** | **GC content** | **Total bases** |
| --- | --- | --- | --- |
| **P1** | 24,126,591 | 47.11% | 7,237,977,400 |
| **P2** | 22,785,112 | 46.89% | 6,835,533,600 |

**Figure S1:** Heat map of clustering of P1 and P2 meiosis-associated genes.

**
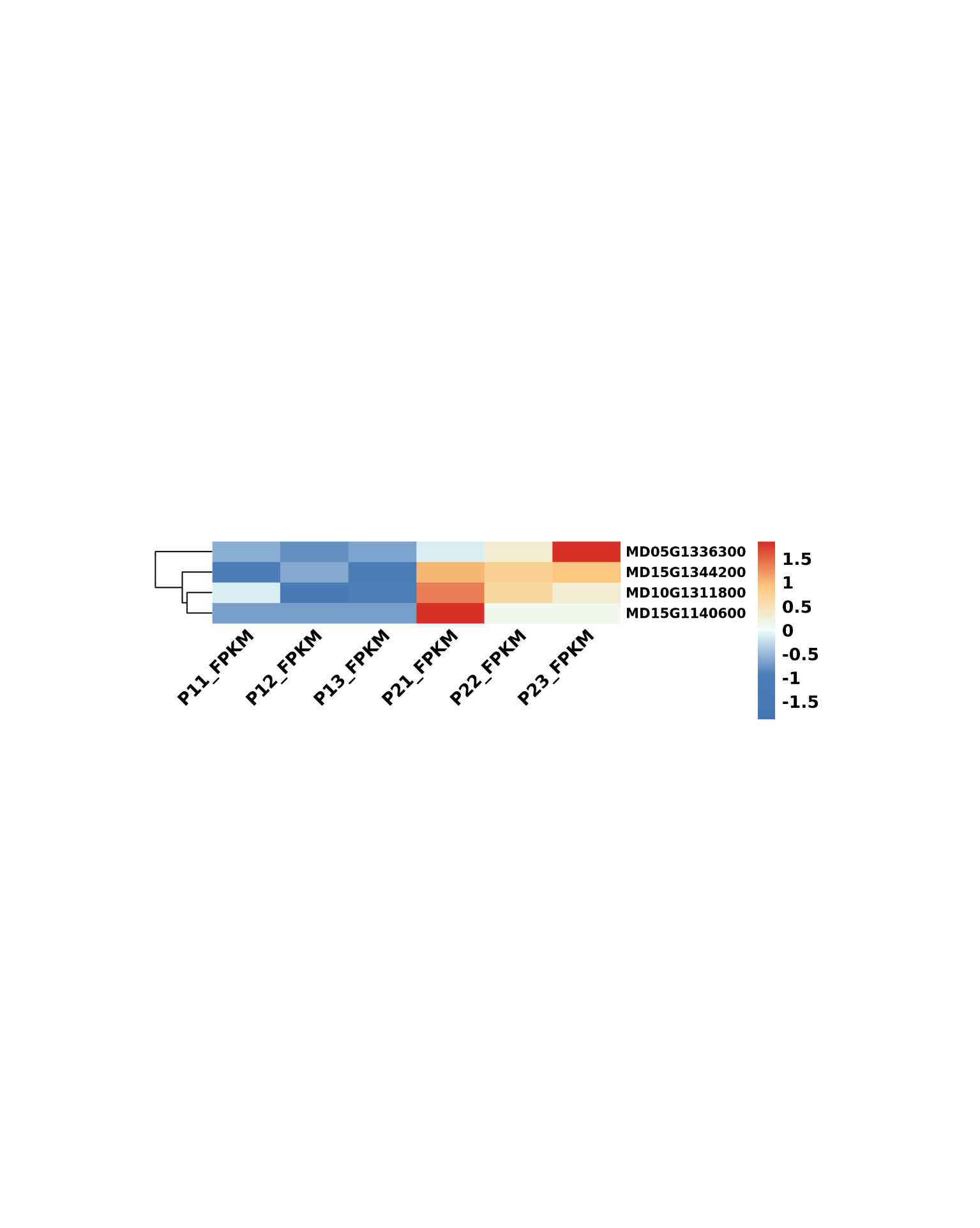
**
